# Evaluating the antifibrotic potential of naringenin, asiatic acid, and icariin using murine and human precision‐cut liver slices

**DOI:** 10.14814/phy2.16136

**Published:** 2024-11-05

**Authors:** Ke Luo, Yana Geng, Dorenda Oosterhuis, Vincent E. de Meijer, Peter Olinga

**Affiliations:** ^1^ Department of Pharmaceutical Technology and Biopharmacy University of Groningen Groningen the Netherlands; ^2^ Department of Surgery, University of Groningen University Medical Center Groningen Groningen the Netherlands

**Keywords:** inflammation, liver fibrosis, natural products, precision‐cut liver slices

## Abstract

Liver fibrosis is an exaggerated wound healing response defined by the excessive accumulation of extracellular matrix. This study investigated the antifibrotic potential of naringenin (NRG), asiatic acid (AA), and icariin (ICA) using murine and human precision‐cut liver slices (PCLS). These natural products have shown promise in animal models, but human data are lacking. In this study, PCLS prepared from male mouse liver tissue (mPCLS), healthy human liver tissue (hhPCLS), and cirrhotic human liver tissue (chPCLS) were cultured for 48 h with varying concentrations of the three compounds. Our findings indicate that NRG reduced collagen type 1 (*COL1A1)* expression in a concentration‐dependent manner in both mPCLS and chPCLS, decreased fibrosis‐related gene expression, and significantly lowered pro‐collagen type 1 (PCOL1A1) levels in the culture medium by 54 ± 21% (mPCLS) and 78 ± 35% (chPCLS). Furthermore, NRG effectively inhibited IL‐1β and TNF‐α in mPCLS and IL‐1β in chPCLS on both gene and protein levels. AA specifically reduced *COL1A1* and PCOL1A1 in chPCLS, while ICA selectively downregulated *Col1a1* and *Acta2* gene expression in mPCLS. This study suggests NRG's potential as an effective antifibrotic agent, warranting further investigation into its mechanisms and therapeutic applications in liver fibrosis.

## 1 INTRODUCTION

Liver fibrosis is an exaggerated wound healing response caused by continuous damage to the liver due to multiple factors, such as viral infections, alcohol misuse, and metabolic disorders (Rockey et al., 2015). Upon chronic liver injury, excessive extracellular matrix protein accumulation leads to scarring of functional liver tissue (Bataller & Brenner, 2005). Liver fibrosis has been known as a reversible dynamic process that can develop slowly over years or decades to cirrhosis (Roehlen et al., 2020). The end stage of fibrosis, cirrhosis, is associated with high mortality and the risk to progress into hepatocellular carcinoma. Liver transplantation is indicated as the only effective curative therapy (Schuppan & Afdhal, 2008). Although significant progress has been made in our understanding of liver fibrosis, treatments for liver fibrosis are scarce, and of limited efficacy. Therefore, there is an urgent need for safe and effective antifibrotic therapies which can prevent or reverse hepatic fibrosis.

Naringenin (NRG), asiatic acid (AA), and icariin (ICA) are three compounds that were reported to exert antifibrotic effects in both in vitro and in vivo liver fibrosis models. NRG is a flavonoid found most abundantly in grapefruit (Felgines et al., 2000). It has been demonstrated to prevent liver damage in animal models induced by alcohol, carbon tetrachloride (CCl_4_), lipopolysaccharide, or heavy metals (Esmaeili & Alilou, 2014; Jayaraman et al., 2012; Ozkaya et al., 2016; Pinho‐Ribeiro et al., 2016). Asiatic acid (AA) is one of the triterpenoid components found in *Centella Asiatica* (Schaneberg et al., 2003). Studies have shown that AA possesses a variety of pharmacological effects on inflammation, oxidation, and wound healing (Pittella et al., 2009; Somboonwong et al., 2012; Yun et al., 2008). Particularly, AA exerted hepatoprotective potential in hepatocytes, hepatic stellate cells, a mouse model induced by D‐galactosamine and lipopolysaccharides, and rat models induced by ethanol or CCl_4_ (Lee et al., 2009; Ma et al., 2009; Tang et al., 2012). Icariin (ICA), a flavonol glucoside, has been isolated from several species of plants belonging to the genus Epimedium (Liu, Li, & Wang, 2006). Much attention has been given to the potential of ICA in osteoporosis prevention, immune system regulation, and improvement of cardiovascular function (Ke et al., 2015; Nian et al., 2009; Shen et al., 2015; Xiong et al., 2016). In addition, ICA treatment protected against renal fibrosis in unilateral ureteral obstruction mice and inhibits TAA‐induced liver fibrosis in rats (Algandaby et al., 2017; Chen et al., 2019).

These abovementioned natural compounds were all reported to possess antifibrotic potency in animal models, but no human data are available so far. Previously, rodent and human ex vivo models of liver fibrosis, precision‐cut liver slices (PCLS), have been successfully used to test putative antifibrotic compounds (Luangmonkong et al., 2017; Olinga & Schuppan, 2013; Westra et al., 2016). The main advantages of PCLS are that all cell types are preserved in their original environment and maintain their intercellular communication. Therefore, we aimed to evaluate the antifibrotic potency of NRG, AA, and ICA in early‐onset of fibrogenesis in mouse PCLS, as well as in early‐onset and end‐stage fibrosis in human PCLS.

## 2 MATERIALS AND METHODS

### 2.1 Ethics statement

The animal experiments were approved by the Animal Ethical Committee of the University of Groningen (DEC 6416AA‐001). The use of human material was approved by the Medical Ethical committee of the university Medical Centre Groningen (UMCG), according to Dutch legislation and the Code of Conduct for dealing responsibly with human tissue in the context of health research, refraining the need of written consent for “further use” of coded‐anonymous human tissue.

### 2.2 Animal livers

Mouse precision‐cut liver slices (mPCLS) were prepared from adult male 10‐week‐old C57BL/6(BL/6), they were housed in a temperature‐ and humidity‐controlled room with a 12 h light/dark cycle, with water and food ad libidum in the Central Animal Facility, UMCG, Groningen, the Netherlands (*n* = 10). The feed (sniff, Germany) was characterized by a high energy density and a medium‐high protein content. Mouse livers were harvested via a terminal procedure performed under isoflurane/O_2_ anesthesia (Nicholas Piramal, London, UK). The experiments were approved by the Animal Ethical Committee of the University of Groningen (DEC 6416AA‐001).

### 2.3 Human livers

Human liver was obtained from surgical excess material of patients. Early‐onset fibrosis was studied in PCLS prepared from donor livers which were regarded as clinically healthy (hhPCLS). End‐stage fibrosis was studied in PCLS prepared from liver tissue of patients with end‐stage liver disease of different etiologies (Table S1) who underwent liver transplantation (chPCLS). Liver tissue was stored in ice‐cold University of Wisconsin (UW) tissue preservation solution until further use the same or next day.

### 2.4 Precision‐cut liver slices (PCLS)

Liver slices were prepared in ice‐cold Krebs–Henseleit buffer supplemented with 25 mM D‐glucose (Merck, Darmstadt, Germany), 25 mM NaHCO_3_ (Merck), 10 mM HEPES (MP Biomedicals, Aurora, US) and saturated with carbogen (95% O_2_/5% CO_2_) using a Krumdieck tissue slicer. In addition, slices were kept on ice‐cold UW solution before culture. PCLS (diameter: 5 mm, thickness: 250 μm) were incubated individually in 1.3 mL of Williams medium E (with L‐glutamine, Invitrogen, Paisley, Scotland) supplemented with 25 mM glucose and 50 μg/mL gentamycin (Invitrogen) at 37°C under continuous supply of 80% O_2_/5% CO_2_ in 12‐well plates while gently shaken to be incubated for 48 h in the presence or absence of different concentrations of the compounds (Westra et al., 2016).

### 2.5 ATP determination

The viability of PCLS was evaluated by assessing ATP content of the slices using a bioluminescence kit (Roche Diagnostics) (de Graaf et al., 2010). ATP values were corrected for the total protein content, estimated by the Lowry assay (Bio‐Rad DC Protein Assay, Hercules, US).

### 2.6 Quantitative real‐time PCR

Total RNA, from pooled three slices, was extracted using the FavorPrep tissue RNA mini kit (FOVORGEN Biotech Corp, Vienna, Austria) according to the manufacturer's instruction and stored at −80°C. RNA concentration was determined using the Synergy HT (Biotek, Swindon, UK) at a wavelength of 260/280. Total RNA (1 μg) was transcribed into cDNA using the Reverse Transcription Kit (Promega, Leiden, the Netherlands) following the manufacturer's instructions and stored at −20°C. Gene expression was determined by either the SYBR (Roche Diagnostics GmbH, Mannheim, Germany) using gene‐specific primers (Supplementary Table S2). The expression of each gene was normalized using the reference gene (GAPDH for mouse samples and 18S for human samples).

### 2.7 Pro‐collagen 1α1 and cytokine measurement

We measured the content of pro‐collagen 1α1 (PCOL1A1) released into the culture medium by PCLS using ELISA kits (mouse PCOL1A1: ab210579, human PCOL1A1: ab210966, Abcam, Cambridge, UK), and cytokine released into the culture medium using Human DuoSet ELISA kits (IL‐1β: DY401 (mouse) and DY201 (human); IL‐6: DY406 (mouse) and DY206 (human); TNF‐α: DY410 (mouse) and DY210 (human); R&D systems, Abingdon, UK). The analyses were performed on medium pooled from three slices from the same group, and it was done according to the manufacturer's protocol.

### 2.8 Statistics

Results are expressed as means ± standard error of the mean (SEM). Student's *t*‐test or ANOVA followed by Fisher's LSD multiple comparisons test were performed using GraphPad Prism 9.0 (La Jolla, CA, USA). A *p*‐value of <0.05 was considered significant when comparing differences between groups.

## 3 RESULTS

### 3.1 Characterization of PCLS

**FIGURE 1:**
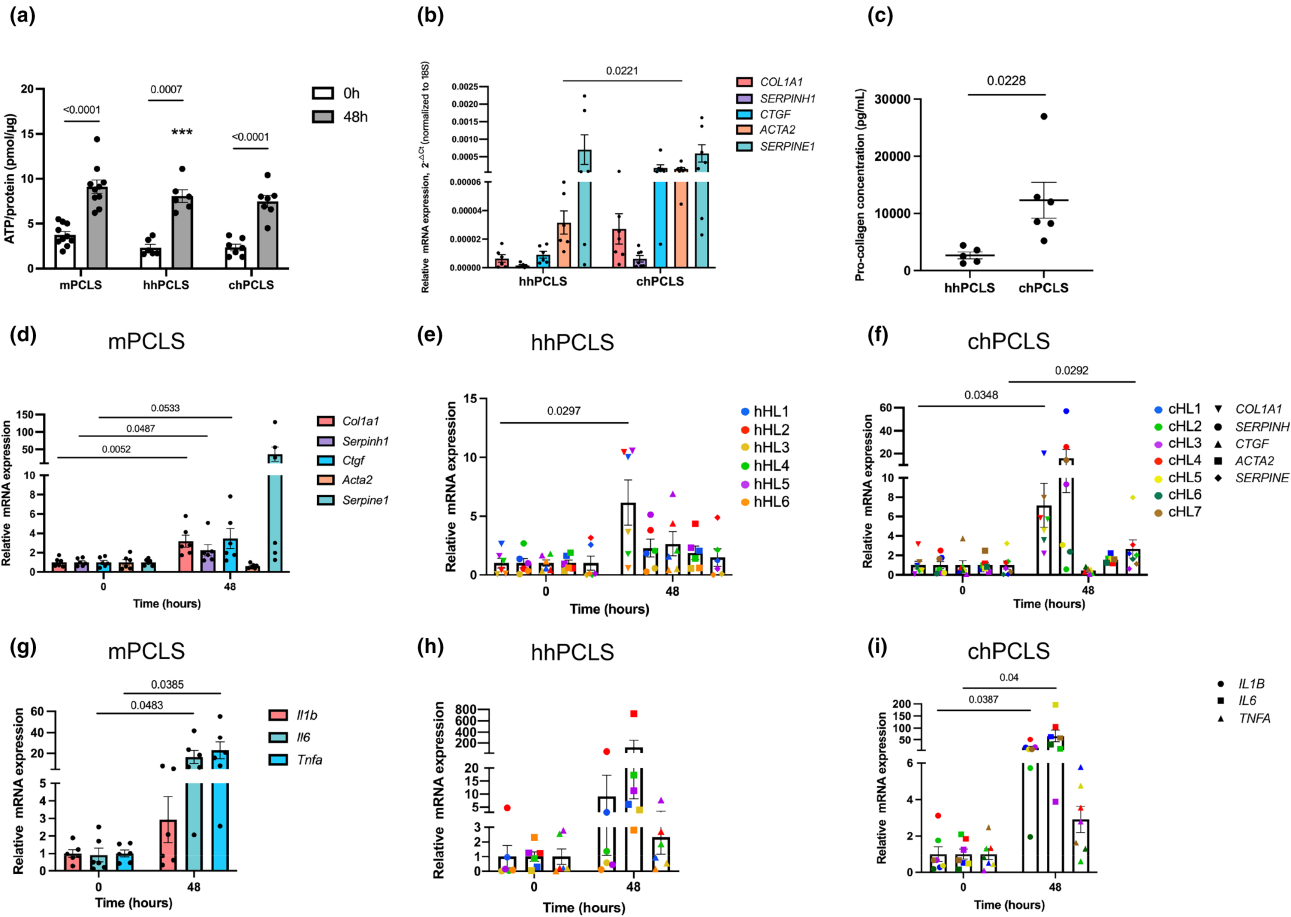
General features of PCLS during incubation. (a) Viability of PCLS at 0 h and after 48 h incubation (ATP/protein ratio); (b) comparison of fibrosis‐related gene expression at 0 h between hhPCLS (*n* = 6) and chPCLS (*n* = 7); (c) comparison of PCOL1A1 release after 48 h incubation between hhPCLS (*n* = 5) and chPCLS (*n* = 6); (d–i) mRNA expression of fibrosis‐related genes in mPCLS (*n* = 6), hhPCLS (*n* = 6), and chPCLS (*n* = 7); (g–i) mRNA expression of inflammation‐related genes in mPCLS (*n* = 6), hhPCLS (*n* = 6), and chPCLS (*n* = 7).

Both mouse and human PCLS remained viable during incubation for up to 48 h, showing an average of 9.1, 8.1, and 7.5 pmol ATP/μg protein in mPCLS, hhPCLS, and chPCLS, respectively (Figure 1a). PCLS of healthy and cirrhotic livers used in this study were investigated by qPCR and ELISA to confirm the healthy and cirrhotic status, respectively. Expression of *Acta2* was 3.8 ± 1.4‐fold (*p* = 0.022) higher in chPCLS than in hhPCLS (Figure 1b), indicating the presence of more activated myofibroblasts in the chPCLS. Moreover, chPCLS secreted 3.6 ± 1.3‐fold (*p* = 0.023) more PCOL1A1 into the culture medium than hhPCLS after 48 h of incubation, further supporting the diseased phenotype of chPCLS (Figure 1c).

### 3.2 Spontaneous fibrosis and inflammation in cultured PCLS

We observed a spontaneous onset of fibrogenesis during culture in all types of PCLS after 48 h of incubation (Figure 1d–i), as revealed by a significant increase of gene expression of *COL1A1* (2.2 ± 0.5 fold in mPCLS, 5.2 ± 1.7 fold in hhPCLS, and 6.1 ± 2.2 fold in chPCLS). Besides, *Serpinh1* and *Ctgf* in mPCLS and *SERPINE1* in chPCLS also increased. When the inflammatory cytokine expressions were measured, *Il6* and *Tnfa* in mPCLS, *IL1B* and *IL6* in chPCLS increased greatly (*p* < 0.05), indicating a more severe inflammatory response in mPCLS and chPCLS during incubation.

### 3.3 Toxicity of naringenin, asiatic acid, and icariin

**FIGURE 2:**
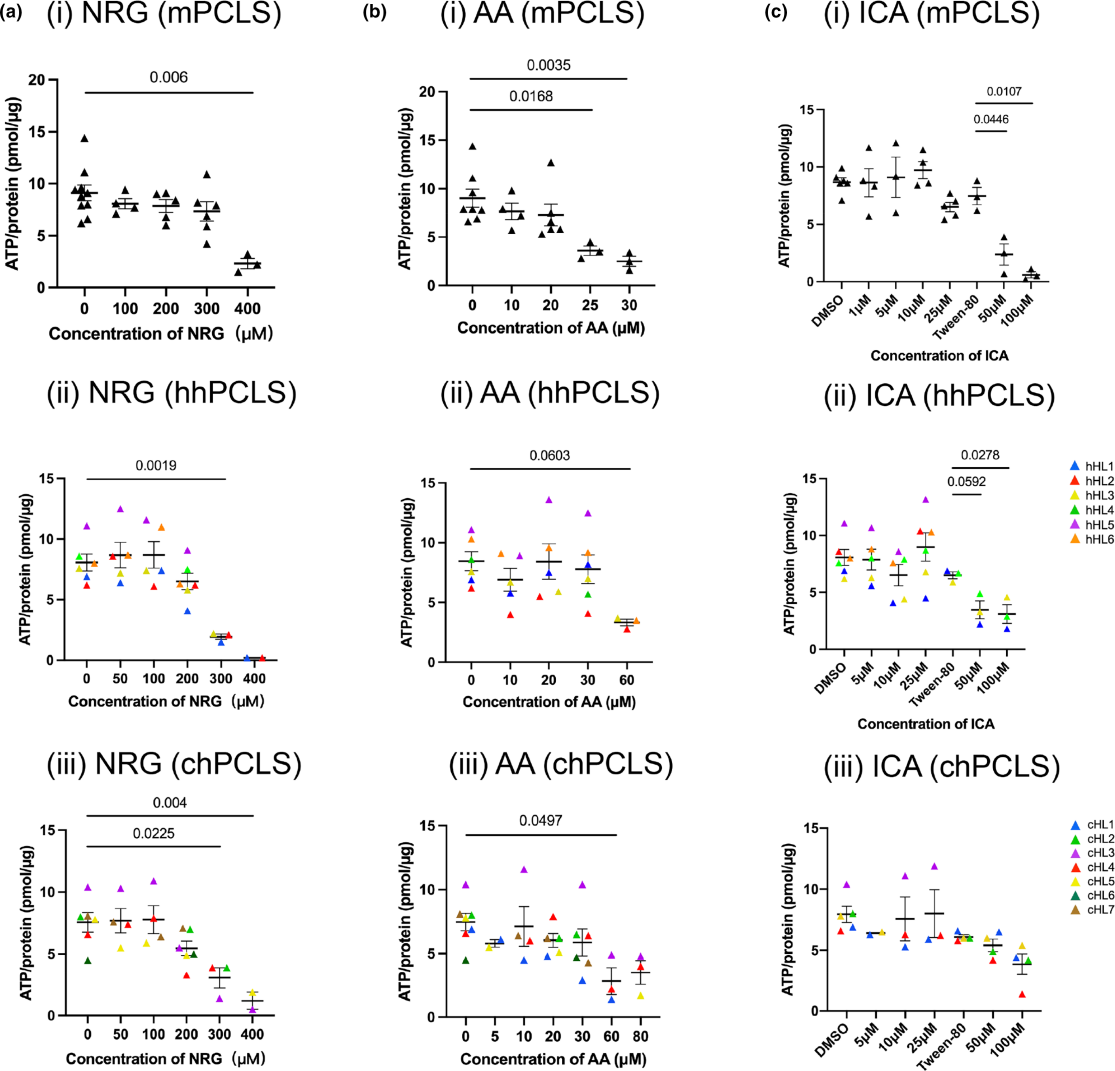
Toxicity of NRG, AA, and ICA. Toxicity of NRG (a), AA (b), and ICA (c) indicated by ATP/protein value in mPCLS (*n* = 3–10), hhPCLS (*n* = 2–6), and chPCLS (*n* = 2–7). In Tween‐80, 50 and 100 μM ICA groups 0.2% of Tween‐80 was added in the culture medium.

Adenosine triphosphate content of PCLS was determined after culturing for 48 hours to study the toxicity of NRG, AA, and ICA. In mPCLS, ATP was significantly reduced by 74.8 ± 14.1% by NRG at 400 μM (Figure 2a, i). In human PCLS, both healthy and cirrhotic, 300 μM of NRG was already toxic and reduced the ATP content significantly (Figure 2a, ii, iii). 25 μM of AA affected the viability of mPCLS significantly as the ATP value dropped to around 50% (Figure 2b, i). Nevertheless, human PCLS seem to be more tolerant to AA, ATP value began to drop when treated with 60 μM of AA (Figure 2b, ii, iii). In mPCLS, 50 μM of ICA significantly influenced the viability (Figure 2c, i). Similar results were observed in hhPCLS (Figure 2c, ii), but interestingly, chPCLS maintained the viability when treated with up to 100 μM ICA (Figure 2c, iii).

### 3.4 Effects of naringenin, asiatic acid, and icariin on inflammation

**FIGURE 3:**
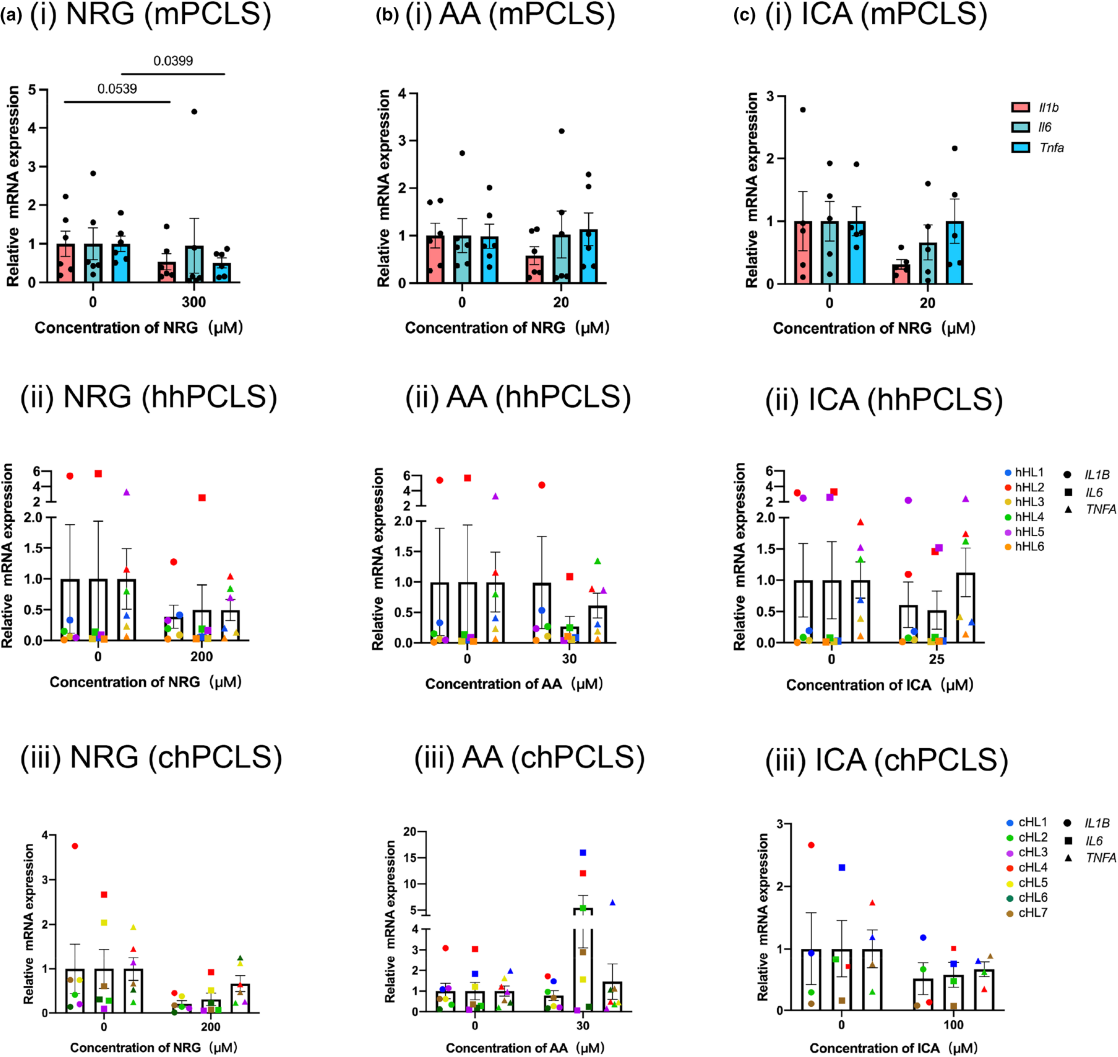
Anti‐inflammatory effects on gene expression. Inflammation‐related gene expression after treated with NRG (a), AA (b), and ICA (c), mPCLS (*n* = 6), hhPCLS (*n* = 6), chPCLS (*n* = 7).

**FIGURE 4:**
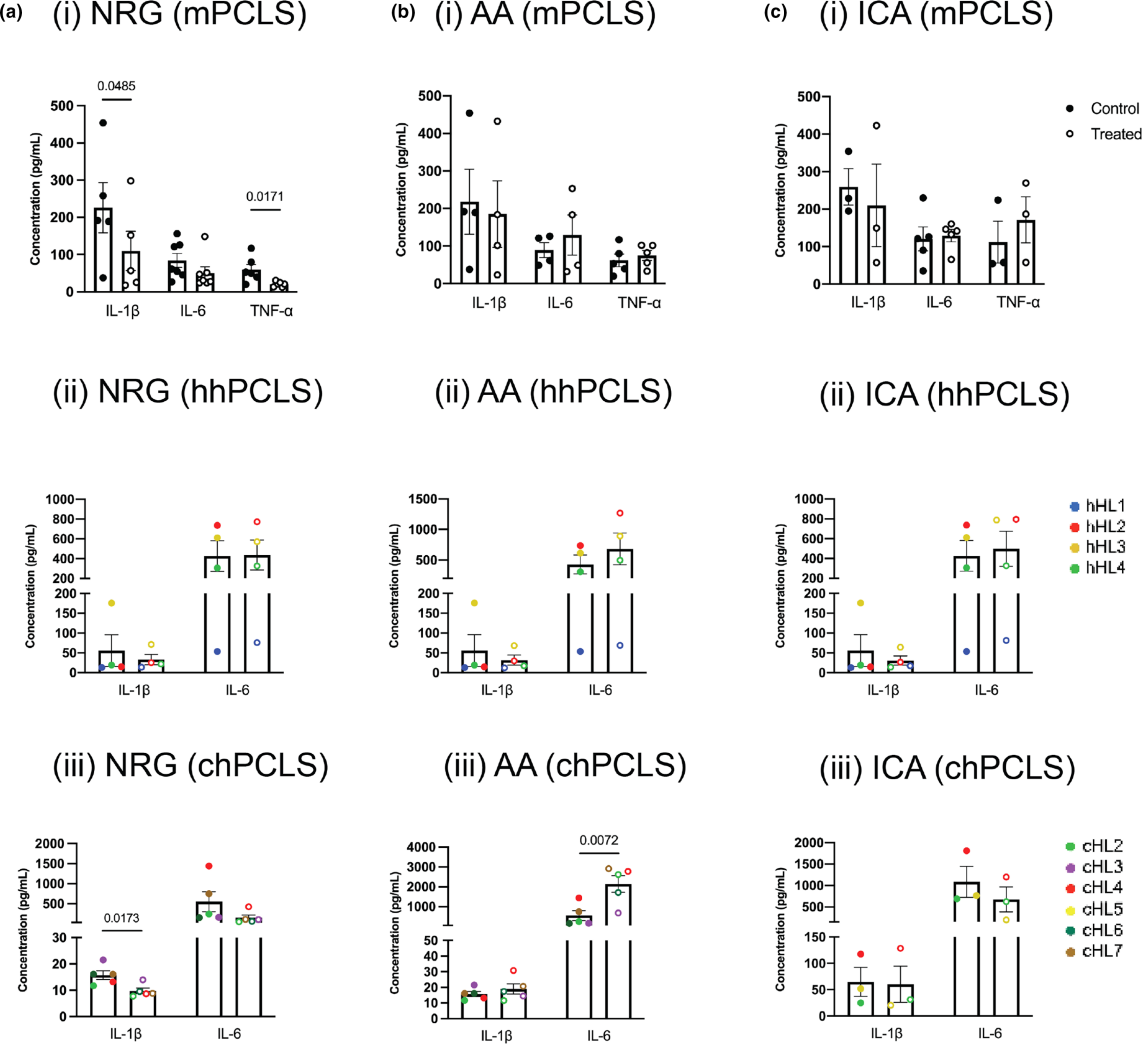
Anti‐inflammatory effects on protein level. The release of IL‐1β, IL‐6, and TNF‐α in the culture medium after treated with NRG (a), AA (b), and ICA (c), mPCLS (*n* = 3–7), hhPCLS (*n* = 4), and chPCLS (*n* = 3–5). TNF‐α was only measured in mPCLS culture medium.

Figures 3 and 4 illustrates the gene expression of three inflammatory makers, *IL1B*, *IL6*, and *TNFA*, and the release of these cytokines in the medium. NRG exhibited better anti‐inflammatory effects than AA and ICA. 300 μM of NRG significantly reduced the gene expression of *Il1b* by 46 ± 18% and *Tnfa* by 49 ± 18% in mPCLS (Figure 3a, i). These effects were also observed on protein levels (Figure 4a, i). In chPCLS, *IL1B* and *IL6* were suppressed by NRG (indicated by the regulation on each liver showed in Supplementary S3), although the effects were not significant, while significantly less IL‐1β was released after the treatment of NRG (Figure 4a, iii). AA and ICA failed to show significant inhibiting effects on these cytokines in all models. In addition, AA increased IL‐6 on both gene and protein levels by 370 ± 185% (*p* = 0.07) (Figure 3b, iii) and 290 ± 60% (*p* = 0.007) (Figure 4b, iii) in chPCLS. In human PCLS medium, TNF‐α was non‐detectable due to the low concentration.

### 3.5 Effects of naringenin, asiatic acid, and icariin on early‐onset of fibrogenesis and end‐stage fibrosis

**FIGURE 5:**
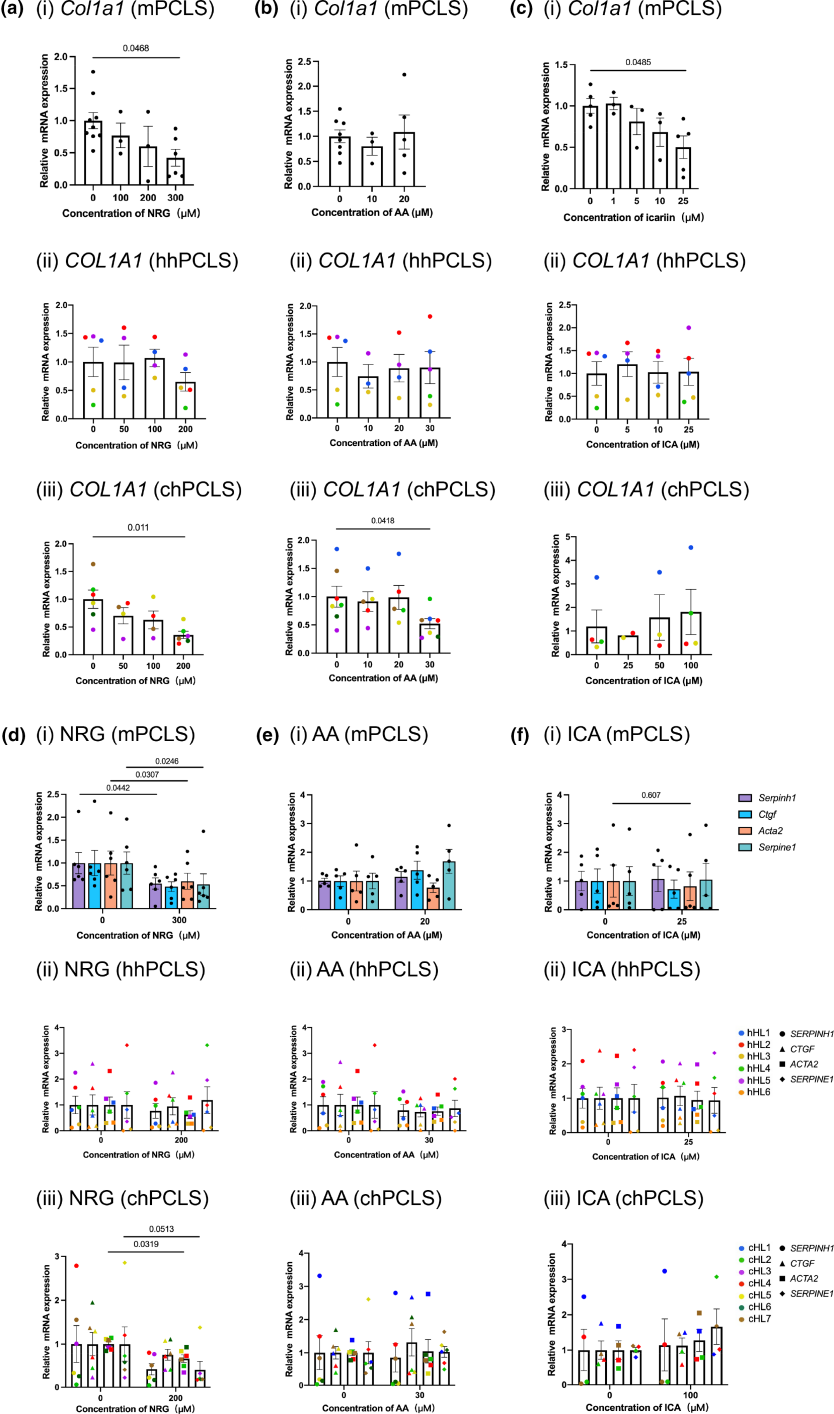
Antifibrotic effects on gene level expression. *COL1A1* gene express after treated with different concentration of NRG (a), AA (b), and ICA (c); Fibrosis‐related gene expression after treated with NRG (d), AA (e), and ICA (f). mPCLS (*n* = 6), hhPCLS (*n* = 6), and chPCLS (*n* = 4–6).

**FIGURE 6:**
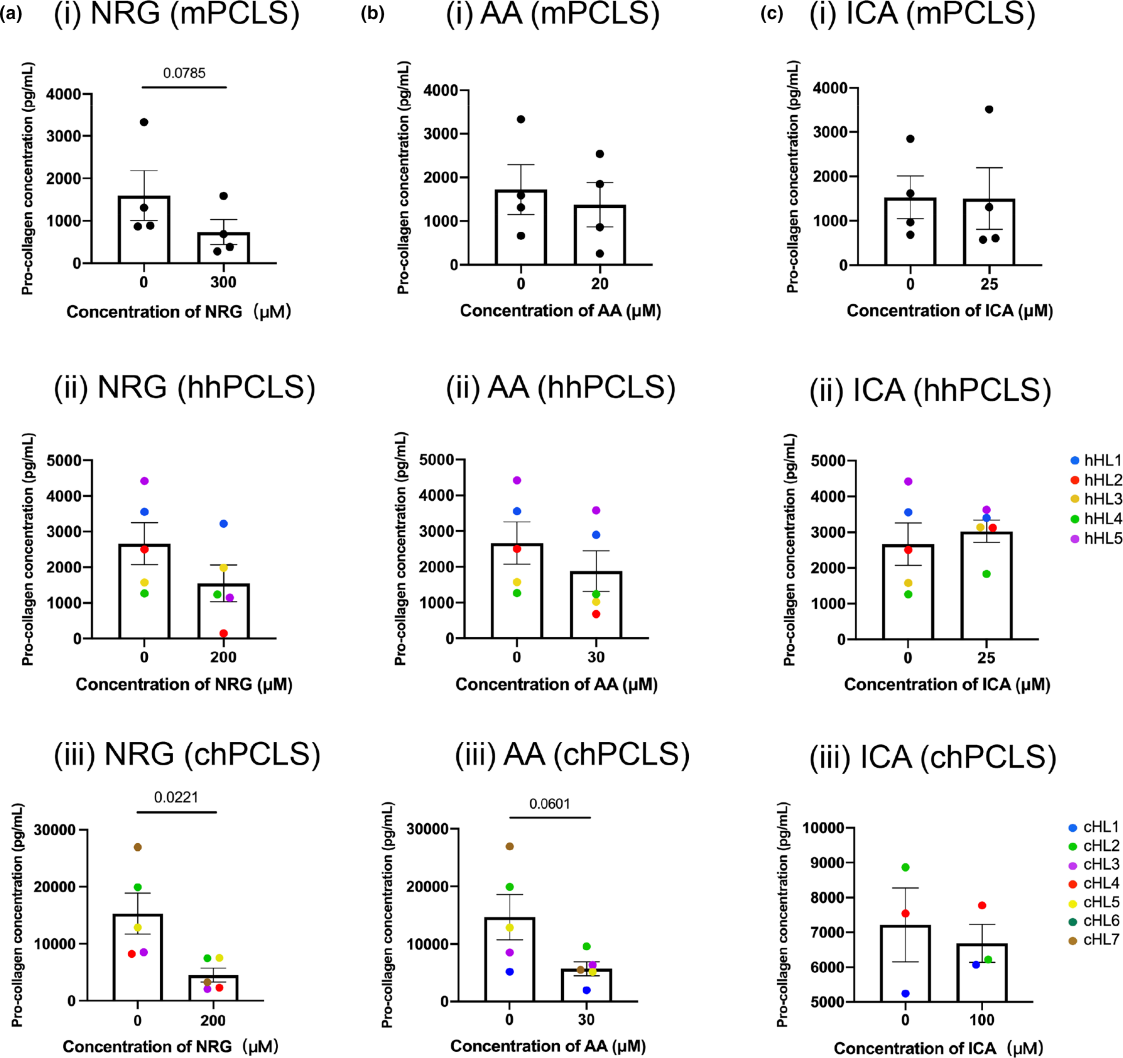
Effects on Pcol1a1 The release of PCOL1A1 in the culture medium after treated with NRG (a), AA (b), and ICA (c), mPCLS (*n* = 4), hhPCLS (*n* = 5), and chPCLS (*n* = 3–5).

To evaluate the antifibrotic effects of NRG, AA, and ICA in PCLS, we first checked how the *COL1A1* gene expression was regulated when treated with different concentrations of these compounds. In mPCLS, NRG and ICA appeared to concentration‐dependently inhibit gene expressions of *Col1a1*, up to 70 ± 25% (*p* = 0.047) and 59 ± 17% (*p* = 0.049), whereas *Col1a1* expression in AA‐treated mPCLS remained unchanged. Unlike in mPCLS, *COL1A1* gene expression was not significantly affected by the treatment of the three compounds in hhPCLS (Figure 5a–c). The effects of the highest non‐toxic concentrations were further investigated by measuring the expression of 4 fibrosis‐related genes, *SERPINH1*, *CTGF*, *ACTA2*, and *SERPINE1* (Figure 5d–f). In mPCLS, 300 μM NRG significantly decreased the expression of *Serpinh1* (45 ± 17%), *Acta2* (45 ± 13%), and *Serpine1* (47 ± 15%). 25 μM ICA downregulated the expression of *Acta2* (25 ± 13%). Nonetheless, no significant regulation by the treatment of compounds was observed in hhPCLS. On protein levels, 300 μM NRG decreased the PCOL1A1 released into the culture medium in mPCLS (54 ± 21%, *p* = 0.08), while the down‐regulation was not observed in hhPCLS (Figure 6a).

Next, we studied whether the antifibrotic effect of the three compounds could also be observed in end‐stage fibrosis using chPCLS. The concentration‐dependent downregulation of *COL1A1* in chPCLS was shown when treated with NRG (Figure 5a), up to 64 ± 18% (*p* = 0.011). Correspondingly, NRG reduced the amount of secreted PCOL1A1 in chPCLS significantly (78 ± 35%, *p* = 0.022) (Figure 6a). When further checking other 4 fibrotic gene expression markers, the treatment with 200 μM NRG results in 35% ± 10% (*p* = 0.032) reduction of *ACTA2* levels and 60 ± 22% (*p* = 0.051) reduction of *SERPINE1* levels. 30 μM AA caused the decrease of *COL1A1* and PCOL1A1 to 47 ± 17% (*p* = 0.041) and 61 ± 23%(*p* = 0.06), but failed to regulate other fibrotic genes. 100 μM ICA didn't show significant effects on *COL1A1*, secreted PCOL1A1, and other four fibrosis‐related genes.

## 4 DISCUSSION

Here, we investigated the antifibrotic effect of the three natural compounds, NRG, AA, and ICA using mouse PCLS, and importantly, for the first time also at the level of individual patients using human PCLS. Results obtained with human PCLS can be interpreted without concerns about possible species differences. In addition, PCLS represent a model of early‐onset and end‐stage fibrosis since they were prepared from healthy and cirrhotic human livers.

### 4.1 Toxicity of naringenin, asiatic acid, and icariin

The toxicity assessment provided insights into the safety profiles of NRG, AA, and ICA. The ATP/Protein assay showed that NRG affected the viability of mouse PCLS at 400 μM and human PCLS at 300 μM, which was consistent with the study on HepG2 cells that NRG did not cause cytotoxicity at concentrations less than 200 μM (Assini et al., 2013). AA became toxic to mouse PCLS at 25 μM, but did not affect the viability of human PCLS below 30 μM. The cytotoxicity of AA on the liver cell line HSC‐T6 was found to start at 40 μM, which aligns with our observations (Tang et al., 2012). The toxicity of icariin has been studied in neither in vivo or in vitro models. Our study revealed that icariin was toxic in both mouse and human healthy tissue at 50 μM, while human cirrhotic PCLS were more tolerant and remained viable up to 100 μM.

### 4.2 Anti‐inflammatory effects of naringenin, asiatic acid, and icariin

Inflammation is a key component and contributor to hepatic wound healing and fibrogenic responses (Seki & Schwabe, 2015). IL‐1β, IL‐6, and TNF‐α are three important cytokines that drive inflammatory responses in the liver. IL‐1β, as one of the most powerful pro‐inflammatory cytokines, participates in toxic, ethanol, and NASH‐induced fibrosis (Gieling et al., 2009; Miura et al., 2010; Petrasek et al., 2012). TNF‐α is another highly pro‐inflammatory cytokine. Effects of TNF‐α are diverse, contributing to hepatocyte apoptosis, immune cell activation, and HSC activation (Tarrats et al., 2011). IL‐6 is a pleiotropic cytokine, exerting a variety of effects on inflammation, liver regeneration, and defends against infections by regulating adaptive immunity (Naseem et al., 2018).

NRG has been reported to exert a potent anti‐inflammatory activity in the liver through the activation of hepatic sirtuin‐1 (SIRT‐1) (Hua et al., 2021) and inhibition of nuclear factor kappa B (NF‐κB) signaling pathway (Chtourou et al., 2015; Jayaraman et al., 2012; Wang et al., 2020). NRG showed an anti‐inflammatory effect at 200 μM in mouse liver by inhibiting IL‐1β and TNF‐α on both gene and protein levels, but not IL‐6, which is consistent with the results in a cholesterol‐fed mice model that NRG prevented hepatic inflammation by inhibiting expression of *Il1b*, *Tnfa*, *Ccl2*, *Ccl3*, and except *Il6* (Wang et al., 2020). In healthy human liver, 200 μM NRG failed to affect the cytokines, while in chPCLS, NRG of the same concentration inhibited the IL‐1β release. These results indicated that the anti‐inflammatory potential of NRG and it might be associated with the activation status of inflammatory pathways in the slices since we observed a stronger inflammatory response in mouse and cirrhotic human livers than healthy human livers during incubation.

AA did not exert the anti‐inflammatory effect as reported in rats with CCl_4_‐induced liver fibrosis that AA significantly reduced hepatic mRNA expression of *Il1b*, *Il6*, and *Tnfa* (Wei et al., 2018). Unexpectedly, 30 μM AA increased IL‐6 expression on gene and protein levels in chPCLS. IL‐6 can serve as an inflammation promotor and accelerate the fibrosis process, signaling via soluble IL‐6 receptor α. Meanwhile, IL‐6 is also known to be a hepatoprotective cytokine that improves hepatocyte proliferation and protects the liver against various forms of injury (Yamaguchi et al., 2010), signaling via membrane‐bound IL‐6Rα (Scheller et al., 2011), the expression of which is restricted to hepatocytes and leucocyte subtypes (Hibi et al., 1990). This might explain why AA was reported to be anti‐inflammatory and here in the cirrhotic human livers, it increased the IL‐6 expression. The effects of the two IL‐6 signaling pathways may counteract each other in the cirrhotic liver environment. Whether the pro‐IL‐6 effects of AA lead to amelioration or worsening of liver cirrhosis still needs further study.

ICA exerts an effect on inflammatory cytokines in different disease models. In rats with brain dysfunction induced by LPS, it decreased IL‐1β, TNF‐α, and COX‐2 expression in the hippocampus (Guo et al., 2010). In a unilateral ureteral obstruction mouse model, that it suppressed expressions of pro‐inflammatory factors including NF‐κB, COX‐2, and IL‐1β (Chen et al., 2019). Here we demonstrated that in the liver, no significant anti‐inflammatory effects of ICA were observed.

### 4.3 Antifibrotic effects of naringenin, asiatic acid, and icariin

There are many studies indicating the protective effects of NRG in preclinical liver fibrosis models, however, no human data are available until now. NRG was reported to be a Smad3‐specific inhibitor and could suppress the TGF‐*β*1‐induced ECM protein expression in cultured rat HSCs (Liu, Wang, et al., 2006) and CCl_4_‐induced rat liver fibrosis model (Hernandez‐Aquino et al., 2019). NRG also improved liver oxidative and inflammatory status via the downregulation of NF‐κB, as well as collagen accumulation by inhibiting matrix metalloproteinases‐2/9 activities in a rat model of high cholesterol‐induced hepatic damage (Chtourou et al., 2015). Here in our study, we observed that NRG not only inhibited COL1A1 at gene and protein levels, and decreased *Ctgf*, *Acta2*, and *Serpine1* expression in mouse PCLS, but also show antifibrotic effects in cirrhotic human livers by suppressing *COL1A1*, PCOL1a1, and gene expression of *ACTA2* and *SERPINE1* significantly, further supporting the therapeutic potency of NRG in liver fibrosis. CTGF can activate the myofibroblasts and stimulates their deposition and remodeling of ECM (Lipson et al., 2012). ACTA2 can be used as a maker of myofibroblast formation (Nagamoto et al., 2000). The level of PAI‐1, encoded by *SERPINE1*, is elevated in fibrotic tissue and inhibits plasmin‐dependent MMP activities, thus, contributing to excessive accumulation of collagen and other ECM proteins (Ghosh & Vaughan, 2012). Taking together the existing data, the down‐regulation of these fibrotic markers, and the fact that NRG at the same time inhibited the inflammatory cytokines in our study, we conclude that NRG might exert its antifibrotic effects through multiple pathways. NRG treatment can regulate HSC activation, ECM remodeling and inhibit the inflammatory signaling pathways, therefore ameliorating liver fibrosis.

A number of studies have reported that treatment with AA was able to induce Smad7, thereby blocking TGF‐β/Smad signaling and ameliorating fibrosis (Tang et al., 2012). And AA can work as a hepatoprotective agent via antimitochondrial stress and the cellular antioxidant system (Lee et al., 2009; Ma et al., 2009). In our study, we found that AA was not able to regulate the fibrotic markers in mouse PCLS and healthy human PCLS, but reduced *COL1A1* expression and PCOL1A1 release in cirrhotic human PCLS. This indicated that compared to NRG, AA might work on more specific pathways to exert antifibrotic effects which might only be activated in the cirrhotic human liver instead of the onset of fibrosis in healthy mouse and human liver during incubation.

ICA was reported to reduce the expressions of *Col1a1* and *Acta2* in the livers from CCl_4_‐challenged mice (Ye et al., 2020) and ameliorate thioacetamide (TAA)‐induced liver fibrosis in rats by inhibiting hepatic *Col1a1* expression and collagen deposition (Algandaby et al., 2017). In mPCLS model, ICA concentration‐dependently inhibited the *Col1a1* expression in mPCLS, but it did not affect the pro‐collagen type I release. In both human PCLS, none of the effects of ICA were found, making it less promising for further human studies.

## 5 CONCLUSION

In summary, our study demonstrated that PCLS is a powerful tool for ex vivo screening of potential antifibrotic drugs. Among the three compounds investigated, NRG exhibited the most promising antifibrotic efficacy. While we observed potential mechanisms related to HSC activation, ECM remodeling, and suppression of pro‐inflammatory cytokines, future studies utilizing next‐generation sequencing and other advanced techniques are necessary to further elucidate the specific pathways involved and confirm these preliminary findings.

## FUNDING INFORMATION

No funding information provided.

## CONFLICT OF INTEREST STATEMENT

No conflicts of interest, financial, or otherwise are declared by the authors.

## Supporting information


Table S1.
Table S2.
