# Supplementary material for: Evaluating the antifibrotic potential of naringenin, asiatic acid, and icariin using murine and human precision‐cut liver slices
Source: Physiol Rep. 2024 Nov 5;12(21):e16136. doi: 10.14814/phy2.16136 (PMC11538472; doi:10.14814/phy2.16136)

Supplementary Table S1 Etiologies of the cirrhotic livers

| Number | Etiology |
| --- | --- |
| cHL1 | Cirrhosis due to alpha-1 antitrypsin deficiency |
| cHL2 | NASH cirrhosis with 25% steatosis |
| cHL3 | NASH cirrhosis |
| cHL4 | NASH cirrhosis |
| cHL5 | Post-alcoholic cirrhosis |
| cHL6 | PSC with biliary cirrhosis |
| cHL7 | NASH cirrhosis |

Supplementary Table S2 List of primers used for qRT-PCR

|  | Gene symbol | Forward sequence | Reverse sequence |
| --- | --- | --- | --- |
|  | *Col1a1* | TGACTGGAAGAGCGGAGAGT | ATCCATCGGTCATGCTCTCT |
|  | *Serpinh1* | AGGTCACCAAGGATGTGGAG | CAGCTTCTCCTTCTCGTCGT |
|  | *Ctgf* | CAAAGCAGCTGCAAATACCA | GGCCAAATGTGTCTTCCAGT |
| Mouse | *Acta2* | ACTACTGCCGAGCGTGAGAT | CCAATGAAAGATGGCTGGAA |
|  | *Serpine1* | GCCAGATTTATCATCAATGACTGGG | GGAGAGGTGCACATCTTTCTCAAAG |
|  | *Il1b* | CTCCACCTCAATGGACAGAA | GCCGTCTTTCATTACACAGG |
|  | *Il6* | GCTACCAAACTGGATATAATCAGGA | CCAGGTAGCTATGGTACTCCAGAA |
|  | *Tnfa* | CTGTAGCCCACGTCGTAGC | TTGAGATCCATGCCGTTG |
|  | *Gapdh* | ACAGTCCATGCCATCACTGC | GATCCACGACGGACACATTG |
|  | *COL1A1* | CAATCACCTGCGTACAGAACGCC | CGGCAGGGCTCGGGTTTC |
|  | *SERPINH1* | GCCCACCGTGGTGCCGCA | GCCAGGGCCGCCTCCAGGAG |
|  | *CTGF* | ACGGCGAGGTCATGAAGAAGAACA | ACTCTCTGGCTTCATGCCATGTCT |
| Human | *ACTA2* | AGGGGGTGATGGTGGGAA | ATGATGCCATGTTCTATCGG |
|  | *SERPINE1* | CACGAGTCTTTCAGACCAAG | AGGCAAATGTCTTCTCTTCC |
|  | *IL1B* | ATGATGGCTTATTACAGTGGCAA | GTCGGAGATTCGTAGCTGGA |
|  | *IL6* | ACTCACCTCTTCAGAACGAATTG | CCATCTTTGGAAGGTTCAGGTTG |
|  | *TNFA* | GAGGCCAAGCCCTGGTATG | CGGGCCGATTGATCTCAGC |
|  | *18S* | CGGCTACCCACATCCAAGGA | CCAATTACAGGGCCTCGAAA |

Supplementary Figure S3 Expression of genes regulated by NRG


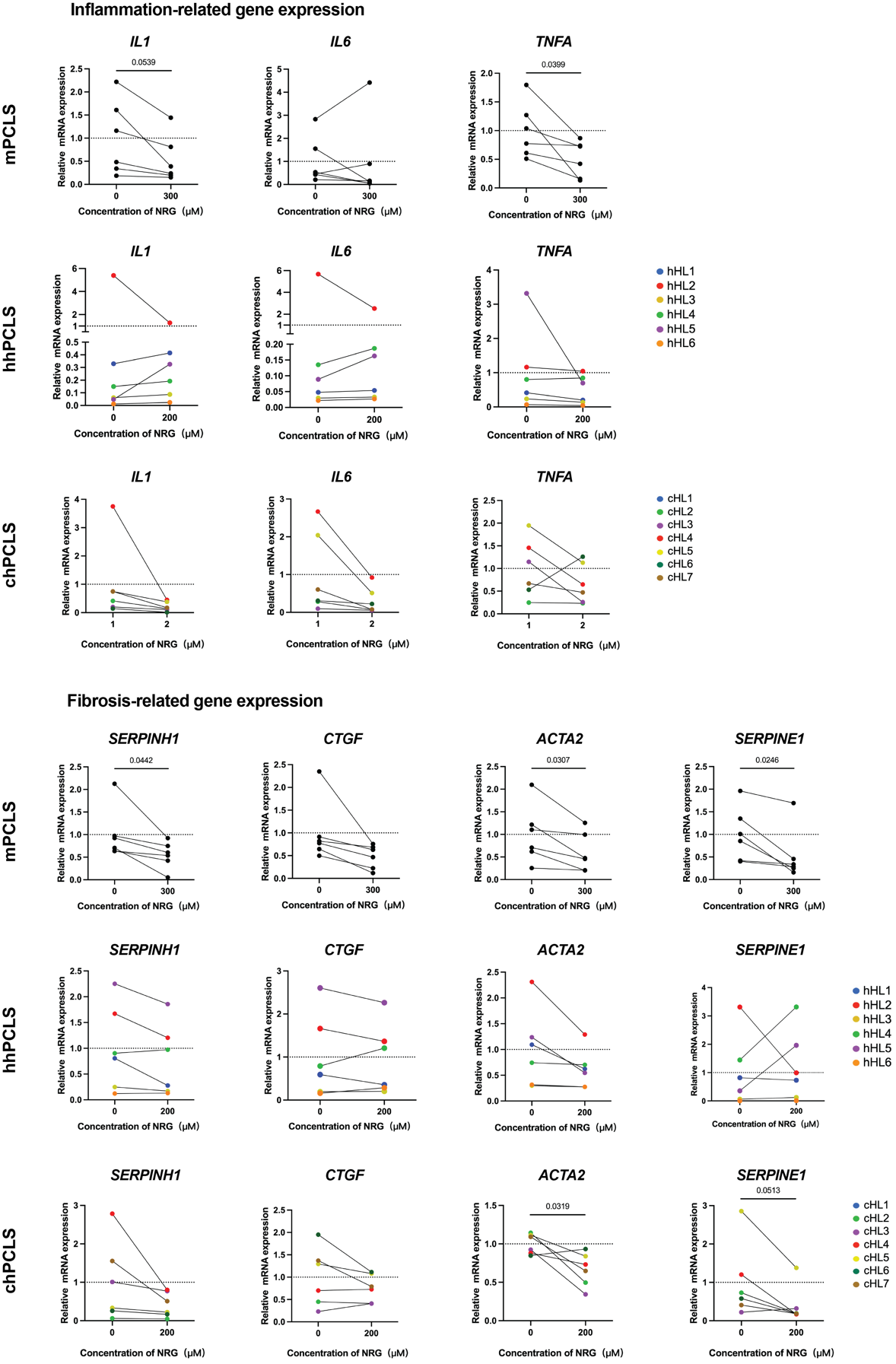

Supplement: Supplementary file 1 — Table S1. Table S2. [file PHY2-12-e16136-s001.docx]
